# Empirical verification of evolutionary theories of aging

**DOI:** 10.18632/aging.101090

**Published:** 2016-10-25

**Authors:** Pavlo Kyryakov, Gomez-Perez Alejandra, Anastasia Glebov, Nimara Asbah, Luigi Bruno, Carolynne Meunier, Tatiana Iouk, Vladimir I. Titorenko

**Affiliations:** ^1^ Department of Biology, Concordia University, Montreal, Quebec H4B 1R6, Canada

**Keywords:** yeast, aging, longevity, evolution, ecosystems

## Abstract

We recently selected 3 long-lived mutant strains of *Saccharomyces cerevisiae* by a lasting exposure to exogenous lithocholic acid. Each mutant strain can maintain the extended chronological lifespan after numerous passages in medium without lithocholic acid. In this study, we used these long-lived yeast mutants for empirical verification of evolutionary theories of aging. We provide evidence that the dominant polygenic trait extending longevity of each of these mutants 1) does not affect such key features of early-life fitness as the exponential growth rate, efficacy of post-exponential growth and fecundity; and 2) enhances such features of early-life fitness as susceptibility to chronic exogenous stresses, and the resistance to apoptotic and liponecrotic forms of programmed cell death. These findings validate evolutionary theories of programmed aging. We also demonstrate that under laboratory conditions that imitate the process of natural selection within an ecosystem, each of these long-lived mutant strains is forced out of the ecosystem by the parental wild-type strain exhibiting shorter lifespan. We therefore concluded that yeast cells have evolved some mechanisms for limiting their lifespan upon reaching a certain chronological age. These mechanisms drive the evolution of yeast longevity towards maintaining a finite yeast chronological lifespan within ecosystems.

## INTRODUCTION

In theory, living organisms can avoid age-related death for a potentially unlimited period of time [1, 2]. This is because from the point of view of thermodynamics living organisms are open self-organizing systems, i.e. they can use exogenous energy to resist a progressive increase in entropy and the resulting molecular damage and disorder as they age [1, 2]. Yet, it is well known that organismal lifespan 1) has a limit that is unique to each species; and 2) varies drastically between different species [3–6]. Since late XIX century, numerous evolutionary theories of aging have been proposed in an attempt to resolve this paradox [1–6]. Theories of programmed aging postulate that the evolutionary force actively restricts organismal lifespan at a certain age distinctive for each species [2, 5–17], whereas theories of non-programmed aging assume that lack of such evolutionary force passively restrains organismal lifespan at a species-specific age [2, 4–6, 15–17]. These two groups of evolutionary aging theories are discussed below.

The first evolutionary theory of aging, known as the theory of programmed death, was developed by August Weismann. According to this theory, natural selection resulted in the preferential reproduction of those members of a particular species that are able to die when they reach a certain age, which is unique to this species [5, 6, 17, 18]. By undergoing a ″programmed″ death at such species-specific age, older members of this species are eliminated from a competition with their younger counterparts for natural resources [5, 6, 17, 18]. In the programmed death theory, the evolutionary advantage to having a limited lifespan at a species-specific age consists in providing a benefit to survival of a group of individuals by creating a disadvantage to those individuals within the group that has reached such an age [5, 6, 17, 18].

Recent advances in the understanding of molecular mechanisms underlying cellular aging and organismal longevity marked a Renaissance period in developing evolutionary theories of programmed aging and age-related death [1, 2, 4, 5–5]. These relatively recently developed theories include the following: 1) group selection theory [5, 6, 15, 17]; 2) kin selection theory [5, 6, 15, 17, 19]; 3) evolvability theory [5, 6, 15, 17, 20]; 4) phenoptosis theory [6 - 9, 21]; and 5) altruistic aging theory [12–14, 22–25]. Akin to the theory of programmed death developed by August Weismann [5, 6, 17, 18], all these contemporary evolutionary theories of programmed aging are based on the notion that natural selection resulted in preferential reproduction of those members of various species that have evolved certain active mechanisms for limiting their lifespans in a species-specific fashion and upon reaching a species-specific age [5–9, 12, 15, 17, 19–21].

Recent studies have provided evidence favoring evolutionary theories of programmed aging and age-related death. In particular, it has been shown that cellular aging can be delayed and organismal longevity can be extended by some genetic, dietary and pharmacological interventions that attenuate certain pro-aging signaling pathways that control the rate of aging [26–59]. These pro-aging signaling pathways operate as active mechanisms that (according to evolutionary theories of programmed aging) can limit organismal lifespan at a specific age. It is conceivable therefore that these pathways have evolved to restrict organismal lifespan at a particular age characteristic of each group of evolutionarily distant organisms.

One of the key features of all contemporary evolutionary theories of programmed aging and age-related death is that longevity-extending genetic traits attenuating different pro-aging signaling pathways may or may not reduce early-life fitness; is has been proposed that early-life fitness can only be decreased by those genetic traits that impair the pro-aging signaling pathways essential for the development of fitness early in life [12, 60–73]. Early-life fitness is known to include the following features: 1) metabolic rate under various environmental conditions; 2) growth rate and, in yeast, the ability to utilize alternative carbon sources; 3) physical activity; 4) fecundity - i.e. the efficacies of mating and reproduction (including sporulation in yeast); 5) resistance to fluctuations in temperature, light, humidity and other environmental factors (such as osmolarity fluctuations in yeast); and 6) susceptibility to environmental toxins [12, 60–73]. Until now the effects of various longevity-extending genetic traits on early-life fitness have been analyzed mainly under laboratory conditions in which long-lived mutants of a certain species were growing and undergoing aging alone, in the absence of “wild-type (WT)” individuals of the same species; these WT individuals do not carry any longevity-extending genetic traits and thus do not have lifespan extended beyond a species-specific age [26–51, 53–55, 74, 75]. However, these laboratory conditions do not mimic the process of natural selection within a mixed population of individuals of the same species. Under such conditions of natural selection, different individuals within the population 1) possess different longevity-defining genetic backgrounds; 2) have lifespans at a species-specific age and above it; and 3) compete for nutrients and other environmental resources [73, 76–81].

Unlike the evolutionary theories of programmed aging and age-related death, all evolutionary theories of non-programmed aging posit that organismal lifespan is limited at an age characteristic of each species due to lack of the evolutionary force [2, 4–6, 15–17]. These theories include the following: 1) the mutation accumulation theory [5, 6, 15, 17, 82, 83] and its modified version known as the late-life mortality plateau theory [5, 6, 15, 17, 84]; and 2) the antagonistic pleiotropy theory [5, 6, 15, 17, 85] and its contemporary version called the disposable soma theory [5, 6, 15, 17, 86 - 88]. Both, the mutation accumulation theory and the late-life mortality plateau theory, postulate that natural selection favours alleles of a gene that are beneficial early in organismal life over alleles of the same gene that provide an advantage late in life of this organism [5, 6, 15, 17, 82–84]. Thus, by eliminating gene alleles that are beneficial late in life, natural selection will diminish its power with age of an organism and will limit its lifespan at an age that is unique to each species [5, 6, 15, 17, 82–84]. In contrast, the antagonistic pleiotropy theory and the disposable soma theory assume that alleles of certain genes that are beneficial in early life of an organism exhibit detrimental effects in its late life [5, 6, 15, 17, 85–88]. Because different alleles of these genes display age-related antagonistic effects on several fitness-defining traits of an organism, these genes are called pleiotropic genes. According to both the antagonistic pleiotropy theory and the disposable soma theory, natural selection limits organismal lifespan at an age unique to each species by actively retaining only those alleles of pleiotropic genes that increase early-life fitness and thus reduce fitness at old age [5, 6, 15, 17, 85–88].

Noteworthy, contemporary evolutionary theories of programmed aging and age-related death postulate that organisms of all species possess mechanisms that have been evolved to actively limit their lifespans at a species-specific age [5, 6, 7–9, 12, 15, 17, 19–21]. In contrast, evolutionary theories of non-programmed aging assume that such mechanisms cannot exist, just because organismal lifespan is limited at a species-specific age passively - i.e. due to lack of the evolutionary force [5, 6, 15, 17, 82–88]. It was therefore concluded that the demonstrated ability of certain genetic, dietary and pharmacological interventions to extend lifespan in evolutionarily distant species by targeting mechanisms that actively limit organismal lifespan at a species-specific age [26–59] validates evolutionary theories of programmed aging and invalidates evolutionary theories of non-programmed aging [5 - 17]. However, in all these cases the ability of genetic, dietary and pharmacological interventions to prolong organismal lifespan has been revealed under laboratory conditions. As discussed above, these conditions do not imitate the process of natural selection within a mixed population of same-species individuals having different longevity-defining genetic backgrounds [73, 76–81]. But none of the evolutionary theories of non-programmed aging assumes that in the absence of natural selection (i.e. under laboratory conditions) longevity-extending mutant gene alleles decreasing early-life fitness cannot exist; all these theories only proclaim that such mutant gene alleles will be eliminated from the gene pool of a species under the pressure of natural selection (i.e. in the wild or under field-like laboratory conditions) [5, 6, 15, 17, 82–88]. Furthermore, it seems impossible in the wild or under field-like laboratory conditions to impose any of the currently known longevity-extending dietary or pharmacological interventions (such as caloric restriction [CR], dietary restriction [DR] or aging-delaying chemical compounds) only on some individuals of the same species; thus, it is unlikely that such non-genetic interventions can be used for empirical verification of evolutionary theories of programmed or non-programmed aging.

We have recently conducted the experimental evolution of long-lived yeast species by a lasting exposure to exogenous lithocholic bile acid (LCA) (Gomez-Perez et al., submitted). We selected 3 long-lived mutants capable of sustaining their greatly extended chronological lifespans (CLS) after numerous passages in medium without LCA (Gomez-Perez et al., submitted). The extended longevity of each of these yeast mutants is a dominant polygenic trait caused by mutations in more than two genes (Gomez-Perez et al., submitted). The objective of this study was to use these long-lived yeast mutants for the empirical verification of evolutionary theories of programmed or non-programmed aging. To attain this objective, we investigated if the dominant polygenic trait extending longevity of each of these mutants affects such key features of early-life fitness as the exponential growth rate, efficacy of post-exponential growth, fecundity, and resistance to apoptotic and liponecrotic forms of programmed cell death. We also examined if any of these long-lived mutants can be forced out of an ecosystem by the parental WT strain exhibiting shorter lifespan; these experiments were carried out under laboratory conditions mimicking the process of natural selection within an ecosystem composed of yeast cells having different longevity-defining genetic back-grounds.

## RESULTS

### Dominant polygenic trait extending longevity of each of the 3 long-lived yeast mutants does not affect some key features of early-life fitness and enhance other such features

To empirically verify evolutionary theories of programmed or non-programmed aging, we elucidated if the dominant polygenic trait that extends longevity of each of the 3 selected long-lived yeast mutants affects early-life fitness when each mutant grows and ages alone – i.e. in the absence of a parental WT strain. The follow-ing key features of early-life fitness were measured: the exponential growth rate and efficacy of post-exponential growth, fecundity, and resistance to apoptotic and liponecrotic forms of programmed cell death.

**Figure 1:**
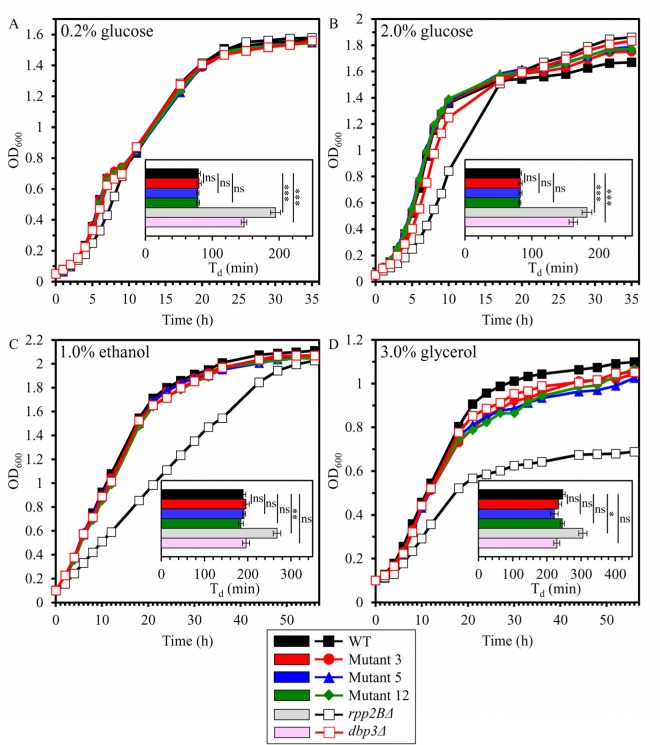
The long-lived mutant strains 3, 5 and 12 do not differ from the parental WT strain in the exponential growth rates and post-exponential growth efficacies in medium initially containing fermentable or non-fermentable carbon source The parental haploid WT strain BY4742, long-lived mutant strains 3, 5 and 12 (each in the BY4742 genetic background), and the single-gene-deletion mutant strains *rpp2BΔ* and *dbp3Δ* (each in the BY4742 genetic background) were cultured in YP medium initially containing 0.2% glucose (**A**), 2.0% glucose (**B**), 1.0% ethanol (**C**) or 3.0% glycerol (**D**). The OD_600_ of cell cultures recovered at different time points was measured. Growth curves are shown; data for growth curves are presented as means (n = 3). For each strain, a doubling time (min) was calculated as T_d_ = (t_2_ – t_1_) × log 2/log (OD_2_/OD_1_), where: t_2_ = a given time point; t_1_ = an earlier time point; OD_2_ = OD_600_ at a given time point; OD_1_ = OD_600_ at an earlier time point. Data for the values of T_d_ are presented as means ± SEM (n = 3; ns, not significant; *p < 0.05; **p < 0.01; ***p < 0.001).

We first assessed if the long-lived mutant strains 3, 5 and/or 12 exhibit altered exponential growth rate and/or efficacy of post-exponential growth in media containing 1) a fermentable carbon source - i.e. glucose at the initial concentration of 0.2% [CR conditions] or 2% [non-CR conditions]; and 2) a non-fermentable carbon source - i.e. ethanol at the initial concentration of 1% or glycerol at the initial concentration of 3%. In these experiments, we used the single-gene-deletion mutant strains *rpp2BΔ* and *dbp3Δ* as controls. Each of these mutant strains is known to exhibit extended replicative lifespan (RLS) and reduced growth rate on 2% glucose [73]. *dbp3Δ* is also known to have prolonged CLS [89]. *rpp2BΔ* lacks a gene encoding ribosomal protein P2 beta, whereas *dbp3Δ* lacks a gene encoding a DEAD-box family protein involved in ribosomal biogenesis [73]. By monitoring the OD_600_ of cell cultures recovered at different time points as a measure of cell growth, we found that the long-lived mutant strains 3, 5 and 12 do not differ from the parental WT strain BY4742 in the exponential growth rates and post-exponential growth efficacies in medium initially containing 0.2% glucose, 2% glucose, 1% ethanol or 3% glycerol (Figures 1A, 1B, 1C and 1D, respectively). Of note, the control strain *rpp2BΔ* exhibited a reduced growth rate in medium initially containing any of these four carbon sources, whereas the control strain *dbp3Δ* displayed a decreased growth rate in medium initially containing 0.2% glucose or 2.0% glucose (Figure 1). Moreover, the control strain *rpp2BΔ* exhibited a significantly reduced efficiency of post-exponential growth in medium initially containing 3% glycerol (Figure 1D).

**Figure 2:**
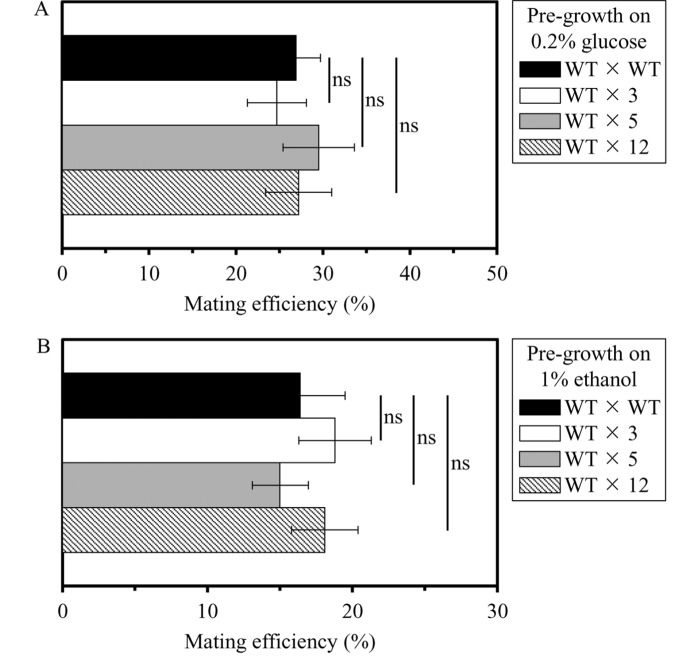
The long-lived mutant strains 3, 5 and 12 do not differ from the parental WT strain in efficacy of their sexual reproduction by mating, a measure of fecundity and a key trait of early-life fitness Yeast cells of mating type *MATa* (i.e. the haploid WT strain BY4741) and mating type *MATα* (i.e. the haploid WT strain BY4742 or the selected long-lived haploid mutant strains 3, 5 or 12, each in the BY4742 genetic background) were pre-grown separately to mid-logarithmic phase in YP medium initially containing 0.2% glucose (a fermentable carbon source; CR conditions) (**A**) or 1% ethanol (a non-fermentable carbon source) (**B**). The efficiency of mating was measured as described in the ″Materials and methods″ section; it was calculated as the number of colonies of *MATa*/*MATα* diploids divided by the sum of *MATa*/*MATα* diploids plus haploid colonies. Data are presented as means ± SEM (n = 3; ns, not significant difference).

We then elucidated if the long-lived mutant strains 3, 5 and/or 12 exhibit altered efficacy of their sexual reproduction by mating, one of the measures of fecundity. In these experiments, yeast cells of mating type *MATa* (i.e. the haploid WT strain BY4741) and mating type *MATα* (i.e. the haploid WT strain BY4742 or the selected long-lived haploid mutant strains 3, 5 or 12, all in the BY4742 genetic background) were pre-grown separately to mid-logarithmic phase in YP medium initially containing 0.2% glucose or 1% ethanol. The efficiency of mating was measured as described in the ″Materials and methods″ section; it was calculated as the number of colonies of *MATa*/*MATα* diploids divided by the sum of *MATa*/*MATα* diploids plus haploid colonies. Crosses between two WT strains of opposite mating types (i.e. the haploid strain BY4741 [*MATa his3D1 leu2D0 met15D0 ura3D0*]) and the haploid strain BY4742 [*MATα his3D1 leu2D0 lys2D0 ura3D0*]) were used as controls. We found that the long-lived mutant strains 3, 5 and 12 do not differ from the parental WT strain BY4742 in efficacy of their sexual reproduction by mating if pre-grown in medium initially containing 0.2% glucose or 1% ethanol (Figures 2A and 2B, respectively).

**Figure 3:**
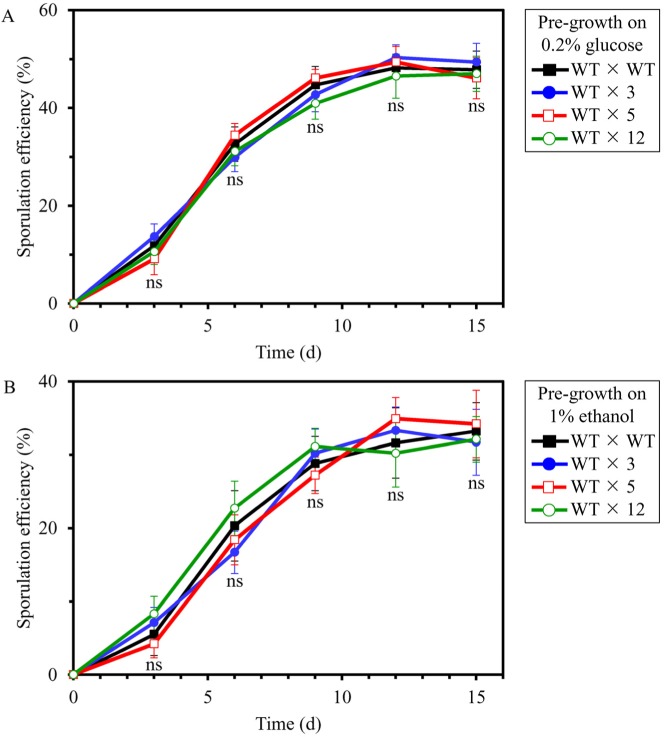
The long-lived mutant strains 3, 5 and 12 do not differ from the parental WT strain in efficacy of their sexual reproduction by sporulation, a measure of fecundity and a key trait of early-life fitness Each of the four diploid strains formed between cells of the haploid WT strain BY4741 (*MATa*) and cells of the haploid WT strain BY4742 (*MATα*) or cells of each of the selected long-lived haploid mutant strains 3, 5 or 12 (each *MATα* in the BY4742 genetic background) were pre-grown to mid-logarithmic phase in YP medium initially containing 0.2% glucose (a fermentable carbon source; CR conditions) (**A**) or 1% ethanol (a non-fermentable carbon) (**B**). The efficiency of sporulation of each of the four diploid strains was measured at various time points since the beginning of a sporulation assay as described in the ″Materials and methods″ section; it was calculated as the percentage of tetrads and dyads produced by a diploid strain, relative to the total number of cells. Data are presented as means ± SEM (n = 3; ns, not significant difference). At each time point, sporulation efficiencies of the WT × 3, WT × 5 and WT × 12 diploid strains were statistically insignificant in comparison with sporulation efficiency of the WT × WT diploid strain.

We then investigated if the long-lived mutant strains 3, 5 and/or 12 display altered efficacy of their sexual reproduction by sporulation, another measure of fecundity. In these experiments, each of the four diploid strains formed between cells of the haploid WT strain BY4741 (*MATa his3D1 leu2D0 met15D0 ura3D0*) and cells of the haploid WT strain BY4742 (*MATα his3D1 leu2D0 lys2D0 ura3D0*) or cells of each of the selected long-lived haploid mutant strains 3, 5 or 12 (each in the BY4742 genetic background) were pre-grown to mid-logarithmic phase in YP medium initially containing 0.2% glucose or 1% ethanol. The efficiency of sporulation of each of the four diploid strains was then measured at various time points since the beginning of a sporulation assay as described in the ″Materials and methods″ section; it was calculated as the percentage of tetrads and dyads produced by a diploid strain, relative to the total number of cells. We found that the long-lived mutant strains 3, 5 and 12 do not differ from the parental WT strain BY4742 in efficacy of their sexual reproduction by sporulation when cells of the hybrid each of them formed with the haploid WT strain BY4741 of opposite mating type were pre-grown in medium initially containing 0.2% glucose or 1% ethanol (Figures 3A and 3B, respectively).

**Figure 4:**
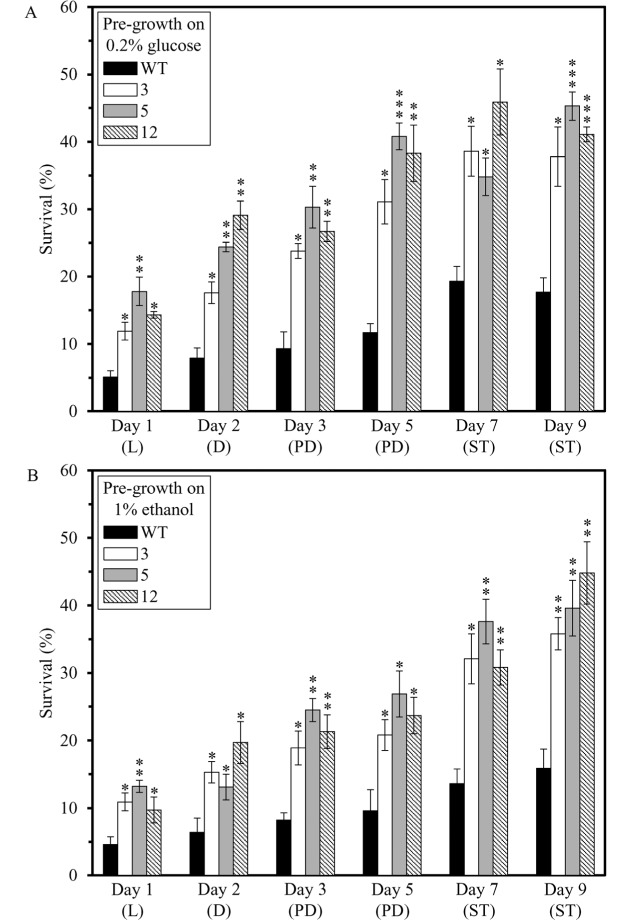
The long-lived mutant strains 3, 5 and 12 exhibit enhanced (as compared to the parental WT strain) susceptibilities to a mitochondria-controlled apoptotic form of cell death, one of the traits of early-life fitness The parental WT strain BY4742 and long-lived mutant strains 3, 5 and 12 (each in the BY4742 genetic background) were cultured in YP medium initially containing 0.2% glucose (a fermentable carbon source; CR conditions) (**A**) or 1% ethanol (a non-fermentable carbon source) (**B**). Cell aliquots were recovered from various growth phases and then treated for 2 h with 2.5 mM hydrogen peroxide to induce mitochondria-controlled apoptosis. The % of viable cells was calculated as described in in the ″Materials and methods″ section. D, diauxic growth phase; L, logarithmic growth phase; PD, post-diauxic growth phase; ST, stationary growth phase. Data originate are presented as means ± SEM (n = 3; *p < 0.05; **p < 0.01; ***p < 0.001).

**Figure 5:**
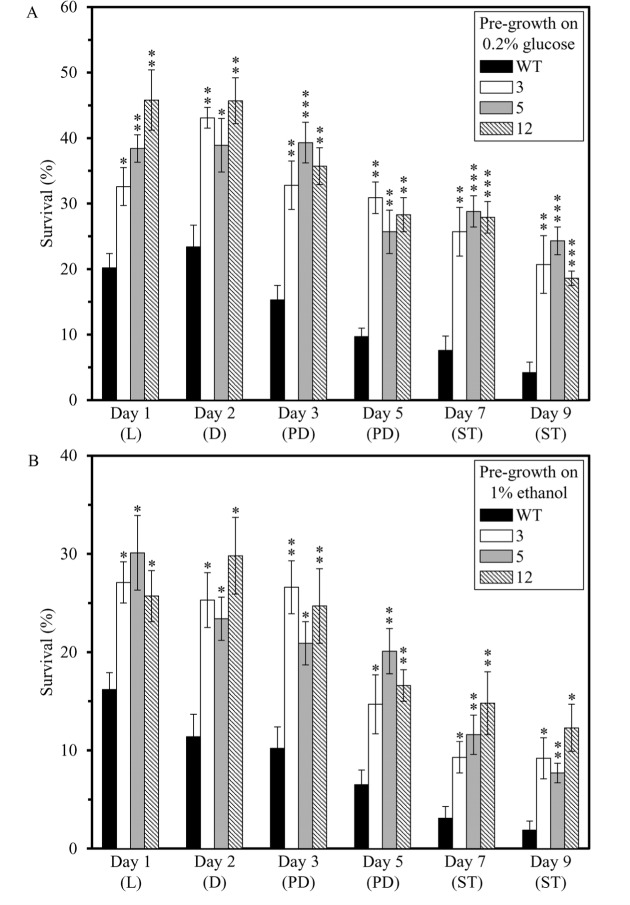
The long-lived mutant strains 3, 5 and 12 exhibit enhanced (as compared to the parental WT strain) susceptibilities to a liponecrotic form of cell death, one of the traits of early-life fitness The parental WT strain BY4742 and long-lived mutant strains 3, 5 and 12 (each in the BY4742 genetic background) were cultured in YP medium initially containing 0.2% glucose (a fermentable carbon source; CR conditions) (**A**) or 1% ethanol (a non-fermentable carbon source) (**B**). Cell aliquots were recovered from various growth phases and then exposed for 2 h to 0.2 mM palmitoleic acid to induce liponecrosis. The % of viable cells was calculated as described in in the ″Materials and methods″ section. D, diauxic growth phase; L, logarithmic growth phase; PD, post-diauxic growth phase; ST, stationary growth phase. Data originate are presented as means ± SEM (n = 3; *p < 0.05; **p < 0.01; ***p < 0.001).

We also assessed if the dominant polygenic trait that extends longevity of each of the 3 long-lived mutant strains affects two other essential aspects of early-life fitness, namely 1) cell susceptibility to a mitochondria-controlled apoptotic form of death triggered by a brief exposure to exogenous hydrogen peroxide [48, 90 - 99]; and 2) cell susceptibility to a ″liponecrotic″ form of death elicited by a short-term exposure to exogenous palmitoleic acid [48, 100 - 103]. We found that the long-lived mutant strains 3, 5 and 12 exhibit enhanced (as compared to the parental WT strain BY4742) susceptibilities to 1) mitochondria-controlled apoptotic death of yeast cells pre-grown in media initially containing 0.2% glucose or 1% ethanol (Figures 4A and 4B, respectively); and 2) liponecrotic death of yeast cells pre-grown in media initially containing 0.2% glucose or 1% ethanol (Figures 5A and 5B, respectively).

Of note, our recent study revealed that the long-lived mutant strains 3, 5 and 12 show enhanced resistance to chronic oxidative, thermal and osmotic stresses (Gomez-Perez et al., submitted). Akin to cell susceptibili-ty to apoptotic and liponecrotic forms of cell death, such resistance to acute stresses is one of the key traits of early-life fitness [12, 48, 58, 61, 65, 99, 100, 104 - 109].

In sum, findings presented in this section and elsewhere (Gomez-Perez et al., submitted) imply that the dominant polygenic traits extending longevities of the long-lived mutant strains 3, 5 and 12 do not affect such key features of early-life fitness as the exponential growth rate, efficacy of post-exponential growth and fecundity. Moreover, these longevity-extending polygenic traits enhance such features of early-life fitness as susceptibility to chronic exogenous stresses, and the resistance to apoptotic and liponecrotic forms of programmed cell death.

### Development and validation of a quantitative assay for assessing the relative fitness of a long-lived mutant strain that competes for nutrients with a parental WT strain

**Figure 6:**
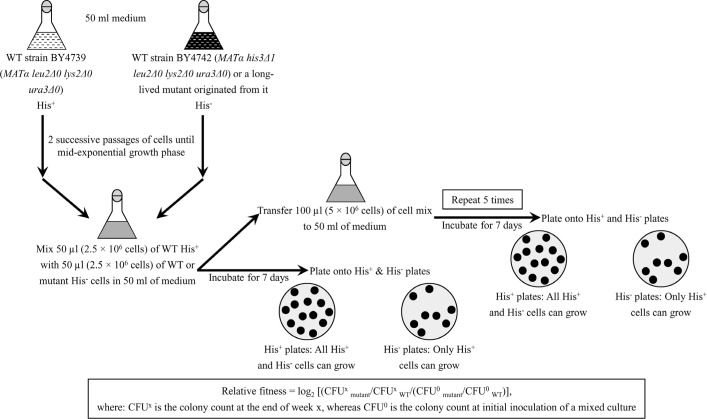
Quantifying the relative fitness of a long-lived mutant strain in a direct competition assay with a parental WT strain His^+^ and His^−^ strains used in the direct fitness competition experiment are first cultured separately in the complete YP medium rich in amino acids, nucleotides and other nutrients until mid-exponential phase. Cells of the His^+^ strain are then mixed with the same number of cells of the His^−^ strain in liquid YP medium. After culturing the cell mixture for 7 days, an aliquot of cell suspension is diluted and plated on a solid YP medium. Following 2 days of incubation, colonies on each plate are replicated onto plates with the synthetic minimal YNB medium without amino acids and nucleotides. One of these plates contains leucine, lysine, uracil and histidine (it is called a His^+^ plate), whereas the other plate contains leucine, lysine and uracil (it is called a ″His^−^″ plate). After 2 days of incubation at 30°C, the number of CFU on ″His^+^″ and ″His^−^″ plates is counted. The relative fitness of each His^+^ strain in a direct competition with the His^−^ is calculated as log_2_ [(CFU^x^_mutant_/CFU^x^_WT_/(CFU° _mutant_/CFU° _WT_)], where: CFU^x^ is the colony count at the end of week x, whereas CFU° is the colony count at initial inoculation of a mixed culture. The direct competition step of culturing a cell mixture for 7 days in liquid YP medium was repeated 6 times.

To investigate if the dominant polygenic trait that extends longevity of each of the 3 selected long-lived yeast mutants influences the relative fitness of the mutant when it competes for nutrients and other environmental resources with a parental WT strain, we developed a direct competition assay. In this assay (Figure 6), the WT strains BY4739 (*MATα leu2D0 lys2D0 ura3D0*) and BY4742 (*MATα his3D1 leu2D0 lys2D0 ura3D0*), the single-gene-deletion mutant strain *dbp3Δ* (*MATα his3(1 leu2(0 lys2(0 ura3(0 dbp3(::kanMX4*) in the BY4742 genetic background, and the long-lived mutant strains 3, 5 and 12 (each in the BY4742 genetic background) were grown separately in the liquid nutrient-rich YP medium initially containing 0.2% glucose, 2% glucose or 1% ethanol as carbon source until mid-exponential phase. The single-gene-deletion mutant strain *dbp3Δ* was used as a control mutant strain because it is known to exhibit 1) extended CLS [89] and RLS [73]; 2) a decreased growth rate on 0.2% glucose (see above), 2% glucose [73] and 1% ethanol (see above); and 3) a reduced relative fitness when it is co-cultured with a parental WT strain in medium initially containing 2% glucose [73, 89]. Cells of the WT strain BY4739 were mixed with the same number of cells of BY4742, *dbp3Δ* or a selected long-lived mutant strain (i.e. mutant strain 3, 5 or 12) in liquid YP medium initially containing 0.2% glucose, 2% glucose or 1% ethanol as carbon source. After culturing the cell mixture for 7 days, an aliquot of cell suspension was diluted and plated on solid YP medium supplemented with 2% glucose. Following 2 days of incubation, colonies on each plate were replicated onto plates with the synthetic minimal YNB medium without amino acids and nucleotides supplemented with 2% glucose. One of these plates contained leucine, lysine, uracil and histidine (hereafter it is called a ″His^+^″ plate), whereas the other plate contained leucine, lysine and uracil (hereafter it is called a ″His^−^″ plate). After 2 days of incubation at 30°C, the number of CFU on ″His^+^″ and ″His^−^″ plates was counted. The relative fitness of each His^+^ strain (i.e. the BY4742, *dbp3Δ*, 3, 5 or 12 strain) in direct competition with the His^−^ strain BY4739 was calculated as log_2_ [(CFU^×^_mutant_/CFU^×^_WT_/(CFU° _mutant_/CFU° _WT_)], where: CFU^x^ is the colony count at the end of week ×, whereas CFU° is the colony count at initial inoculation of a mixed culture (Figure 6). In every experiment for measuring relative fitness, the direct competition step of culturing a cell mixture for 7 days in liquid YP medium was repeated 6 times.

**Figure 7:**
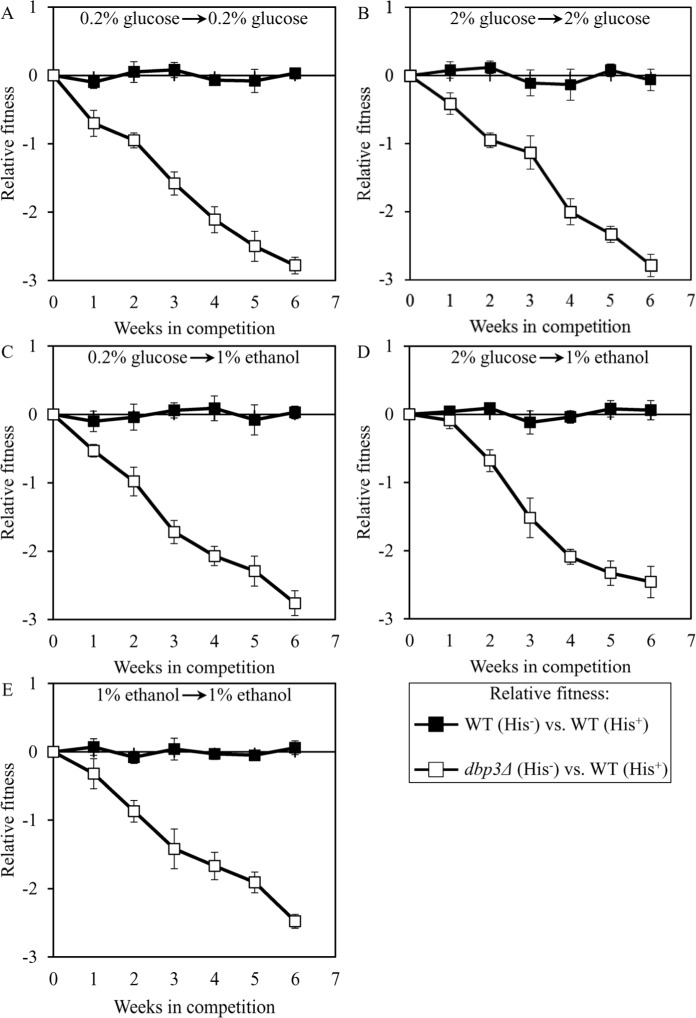
Validation of the developed assay for quantifying the relative fitness of a long-lived mutant strain in direct competition with a parental WT strain The WT strains BY4742 (His^−^) and BY4739 (His^+^, but otherwise isogenic to BY4742) were cultured separately in the complete YP medium containing 0.2% glucose, 2% glucose or 1% ethanol glucose until mid-exponential phase. Another pair of strains whose relative fitness was measured, namely the long-lived mutant strain *dbp3Δ* (His^−^; is isogenic to BY4742) and the WT strain BY4739 (His^+^), was also cultured separately in YP medium containing 0.2% glucose 2% glucose or 1% ethanol glucose until mid-exponential phase. Cells of the His^+^ strain were mixed with the same number of cells of the His^−^ strain and then co-cultured for 7 days in liquid YP medium initially containing different carbon sources. Cells of the His^−^ and His^+^ strains pre-cultured separately on 0.2% glucose were subjected to direct fitness competition by being cultured together on 0.2% glucose (**A**) or 1% ethanol (**C**). Cells of the His^−^ and His^+^ strains pre-cultured separately on 2% glucose were subjected to direct fitness competition by being cultured together on 2% glucose (**B**) or 1% ethanol (**D**). Cells of the His^−^ and His^+^ strains pre-cultured separately on 1% ethanol were subjected to direct fitness competition by being cultured together on 1% ethanol (**E**). After culturing the cell mixture for 7 days, an aliquot of cell suspension was used to measure the relative fitness of the His^+^ strain in direct competition with the His^−^ strain (as described in “Materials and Methods”). The direct fitness competition step of culturing a cell mixture for 7 days in liquid YP medium was repeated 6 times.

To validate this assay in a control experiment, we compared the fitness of the WT strain BY4742 (His^−^; the parental strain of the long-lived mutant strains 3, 5 and 12) to that of the WT strain BY4739 (His^+^, but otherwise isogenic to BY4742). We found that even after 6 consecutive 7-days incubations BY4742 (His^−^) exhibits similar relative fitness in a direct competition assay with BY4739 (His^+^) co-cultured in YP medium initially containing the following carbon source: 1) 0.2% glucose, after cell transfer from 0.2% glucose (Figure 7A); 2) 2% glucose, after cell transfer from 2% glucose (Figure 7B); 3) 1% ethanol, after cell transfer from 0.2% glucose (Figure 7C); 4) 1% ethanol, after cell transfer from 2% glucose (Figure 7D); or 5) 1% ethanol, after cell transfer from 1% ethanol (Figure 7E). Based on these findings, we concluded that the developed direct competition assay outlined in Figure 6 accurately reproduces the expected equal fitness of each of the two WT strains used, i.e. BY4739 (His^+^) and BY4742 (His^−^). Moreover, this assay also accurately reproduces the reduced fitness [73, 89] of the mutant strain *dbp3Δ* (which is isogenic to the WT strain BY4742) in direct competition with the parental WT strain BY4739 (His^+^) (Figure 7).

### Dominant polygenic trait extending longevity of each of the 3 long-lived yeast mutants decreases its relative fitness under some laboratory conditions

**Figure 8:**
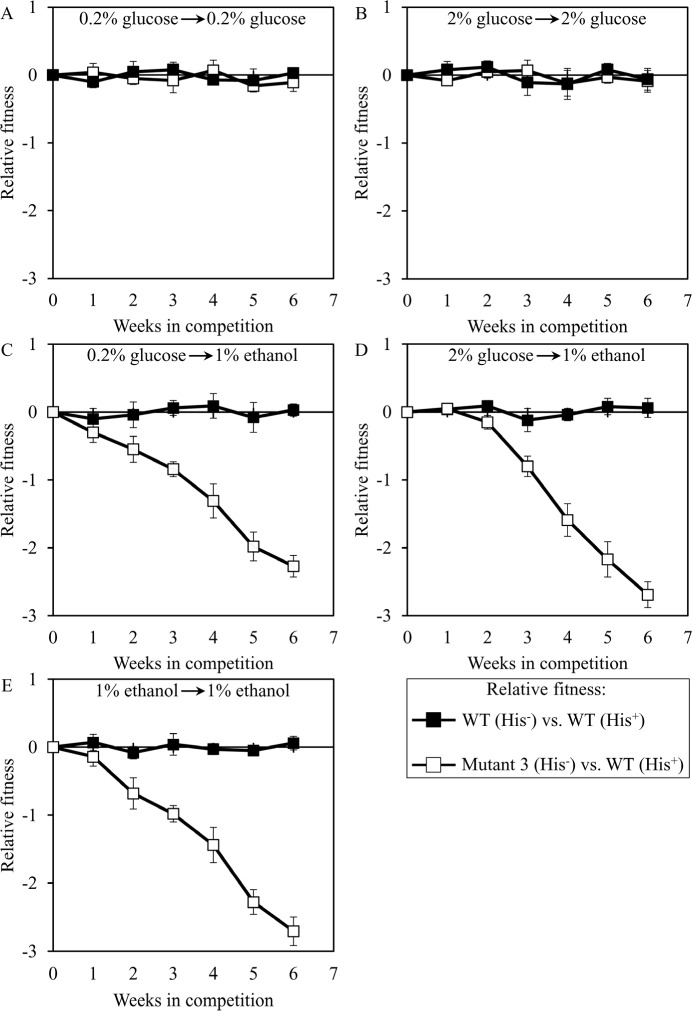
Dominant polygenic trait extending longevity of the long-lived yeast mutant 3 decreases its relative fitness in medium initially containing 1% ethanol The WT strains BY4742 (His^−^) and BY4739 (His^+^, but otherwise isogenic to BY4742) were cultured separately in the complete YP medium containing 0.2% glucose, 2% glucose or 1% ethanol glucose until mid-exponential phase. Another pair of strains whose relative fitness was measured, namely the long-lived mutant strain 3 (His^−^; selected during lasting exposure of BY4742 to LCA) and the WT strain BY4739 (His^+^), was also cultured separately in YP medium containing 0.2% glucose 2% glucose or 1% ethanol glucose until mid-exponential phase. Cells of the His^+^ strain were mixed with the same number of cells of the His^−^ strain and then co-cultured for 7 days in liquid YP medium initially containing different carbon sources. Cells of the His^−^ and His^+^ strains pre-cultured separately on 0.2% glucose were subjected to direct fitness competition by being cultured together on 0.2% glucose (**A**) or 1% ethanol (**C**). Cells of the His^−^ and His^+^ strains pre-cultured separately on 2% glucose were subjected to direct fitness competition by being cultured together on 2% glucose (**B**) or 1% ethanol (**D**). Cells of the His^−^ and His^+^ strains pre-cultured separately on 1% ethanol were subjected to direct fitness competition by being cultured together on 1% ethanol (**E**). After culturing the cell mixture for 7 days, an aliquot of cell suspension was used to measure the relative fitness of the His^+^ strain in a direct competition with the His^−^ strain (as described in ″Materials and Methods″). The direct fitness competition step of culturing a cell mixture for 7 days in liquid YP medium was repeated 6 times.

**Figure 9:**
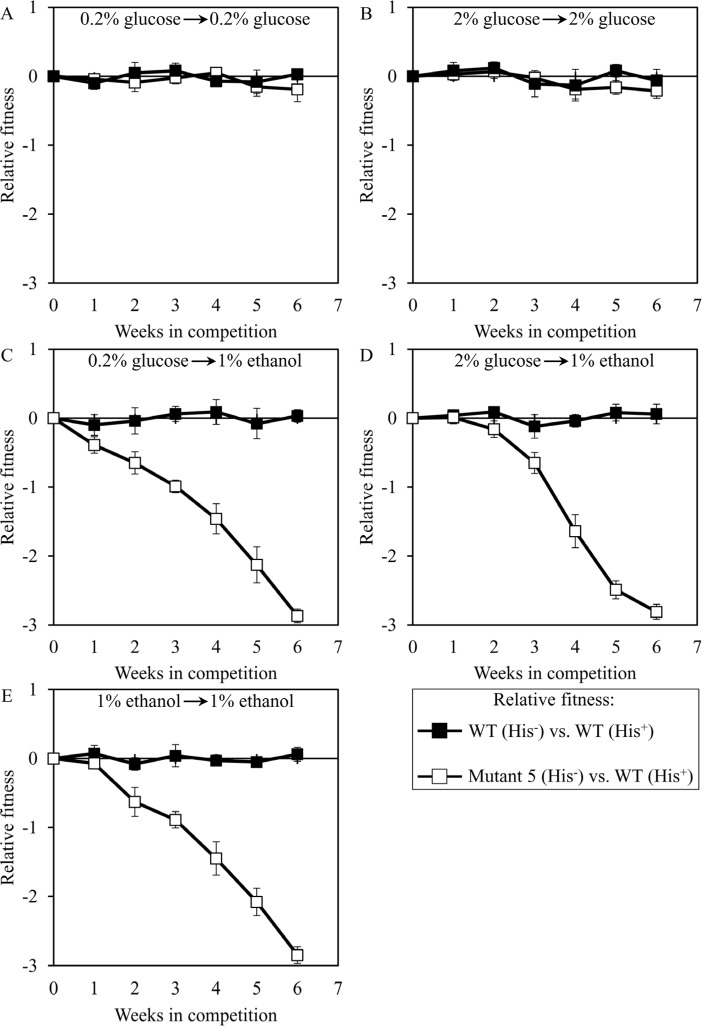
Dominant polygenic trait extending longevity of the long-lived yeast mutant 5 decreases its relative fitness in medium initially containing 1% ethanol The WT strains BY4742 (His^−^) and BY4739 (His^+^, but otherwise isogenic to BY4742) were cultured separately in the complete YP medium containing 0.2% glucose, 2% glucose or 1% ethanol glucose until mid-exponential phase. Another pair of strains whose relative fitness was measured, namely the long-lived mutant strain 5 (His^−^; selected during lasting exposure of BY4742 to LCA) and the WT strain BY4739 (His^+^), was also cultured separately in YP medium containing 0.2% glucose 2% glucose or 1% ethanol glucose until mid-exponential phase. Cells of the His^+^ strain were mixed with the same number of cells of the His^−^ strain and then co-cultured for 7 days in liquid YP medium initially containing different carbon sources. Cells of the His^−^ and His^+^ strains pre-cultured separately on 0.2% glucose were subjected to direct fitness competition by being cultured together on 0.2% glucose (**A**) or 1% ethanol (**C**). Cells of the His^−^ and His^+^ strains pre-cultured separately on 2% glucose were subjected to direct fitness competition by being cultured together on 2% glucose (**B**) or 1% ethanol (**D**). Cells of the His^−^ and His^+^ strains pre-cultured separately on 1% ethanol were subjected to direct fitness competition by being cultured together on 1% ethanol (**E**). After culturing the cell mixture for 7 days, an aliquot of cell suspension was used to measure the relative fitness of the His^+^ strain in a direct competition with the His^−^ strain (as described in ″Materials and Methods″). The direct fitness competition step of culturing a cell mixture for 7 days in liquid YP medium was repeated 6 times.

**Figure 10:**
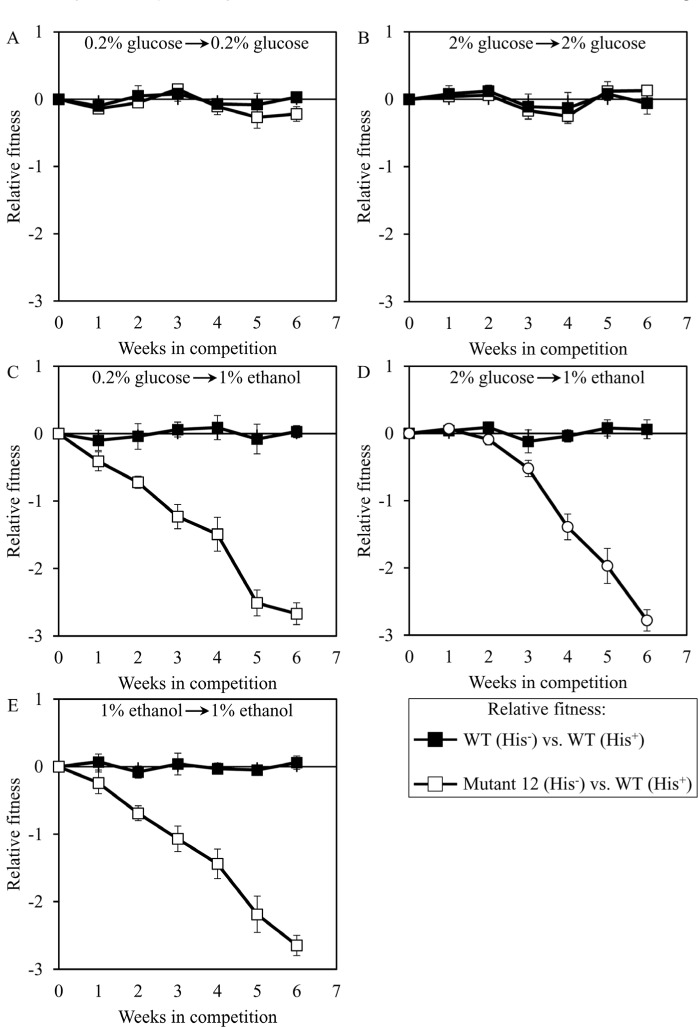
Dominant polygenic trait extending longevity of the long-lived yeast mutant 12 decreases its relative fitness in medium initially containing 1% ethanol The WT strains BY4742 (His^−^) and BY4739 (His^+^, but otherwise isogenic to BY4742) were cultured separately in the complete YP medium containing 0.2% glucose, 2% glucose or 1% ethanol glucose until mid-exponential phase. Another pair of strains whose relative fitness was measured, namely the long-lived mutant strain 12 (His^−^; selected during lasting exposure of BY4742 to LCA) and the WT strain BY4739 (His^+^), was also cultured separately in YP medium containing 0.2% glucose 2% glucose or 1% ethanol glucose until mid-exponential phase. Cells of the His^+^ strain were mixed with the same number of cells of the His^−^ strain and then co-cultured for 7 days in liquid YP medium initially containing different carbon sources. Cells of the His^−^ and His^+^ strains pre-cultured separately on 0.2% glucose were subjected to direct fitness competition by being cultured together on 0.2% glucose (**A**) or 1% ethanol (**C**). Cells of the His^−^ and His^+^ strains pre-cultured separately on 2% glucose were subjected to direct fitness competition by being cultured together on 2% glucose (**B**) or 1% ethanol (**D**). Cells of the His^−^ and His^+^ strains pre-cultured separately on 1% ethanol were subjected to direct fitness competition by being cultured together on 1% ethanol (**E**). After culturing the cell mixture for 7 days, an aliquot of cell suspension was used to measure the relative fitness of the His^+^ strain in a direct competition with the His^−^ strain (as described in ″Materials and Methods″). The direct fitness competition step of culturing a cell mixture for 7 days in liquid YP medium was repeated 6 times.

We used the developed direct competition assay to measure the relative fitness of the long-lived mutant strain 3, 5 or 12 in direct competition with a parental WT strain. Cells of each of these mutant strains were first cultured separately in liquid YP medium containing different concentrations of glucose or ethanol. Cells of each mutant strain were then mixed with the same number of cells of the WT strain BY4739 (His^+^, but otherwise isogenic to the parental WT strain BY4742) and underwent 6 consecutive 7-days incubations together. We found that the dominant polygenic trait extending longevity of the long-lived mutant strain 3, 5 or 12 does not alter its relative fitness in a direct competition assay with the parental WT strain co-cultured in medium initially containing one of the following carbon sources: 1) 0.2% glucose, after cell transfer from 0.2% glucose (Figures 8A, 9A and 10A, respectively); or 2) 2% glucose, after cell transfer from 2% glucose (Figures 8B, 9B and 10B, respectively). In contrast, the dominant polygenic trait extending longevity of the long-lived mutant strain 3, 5 or 12 decreased its relative fitness in a direct competition assay with the parental WT strain co-cultured in medium initially containing 1% ethanol, after cell transfer from any of the following carbon sources: 1) 0.2% glucose (Figures 8C, 9C and 10C, respectively); 2) 2% glucose (Figures 8D, 9D and 10D, respectively); or 3) 1% ethanol (Figures 8E, 9E and 10E, respectively).

Our findings revealed that the conditions of pre-culturing of any of the 3 long-lived mutant strains do not influence the extent of its decreased relative fitness during the subsequent co-culturing with the parental WT strain in medium initially containing 1% ethanol (Figures 8C–8E, 9C–9E, 10C–10E). We therefore concluded that none of these long-lived mutant strains keeps a ″memory″ of conditions under which it has been grown prior to being mixed with the parental WT strain in medium supplemented with 1% ethanol for fitness competition.

## DISCUSSION

Using the 3 long-lived mutant strains selected during experimental evolution under laboratory conditions (Gomez-Perez et al., submitted) in this study we empirically verified evolutionary theories programmed or non-programmed aging. We demonstrate that the dominant polygenic trait extending longevity of each of these mutants does not affect such key features of early-life fitness as the exponential growth rate, efficacy of post-exponential growth, and fecundity (which was assessed by measuring the efficacies of mating and sporulation). These findings provide evidence in support of evolutionary theories of programmed aging and invalidate evolutionary theories of non-programmed aging and age-related death. Indeed, all evolutionary theories of non-programmed aging and age-related death predict that any longevity-extending genetic trait must decrease early-life fitness of an organism if it grows and ages alone, in the absence of WT individuals of the same species; these WT individuals do not carry longevity-extending mutations and thus do not have lifespan extended beyond a species-specific age [2, 4–6, 15–17, 82–88].

This study and our recent findings (Gomez-Perez et al., submitted) show for the first time that a longevity-extending genetic trait can enhance such features of early-life fitness as susceptibility to chronic exogenous stresses, and the resistance to apoptotic and liponecrotic forms of programmed cell death. We have observed this enhancement of some early-life fitness features in the 3 long-lived mutant strains when each of them was growing and undergoing chronological aging in the absence of the parental WT yeast strain (i.e. in the absence of natural selection).

In this study, we also developed and validated a direct competition assay for the measurement of relative fitness under laboratory conditions. This assay mimics the process of natural selection within a mixed population of yeast cells that 1) exhibit different longevity-defining genetic backgrounds; 2) differ in their lifespans if grow as a genetically homogenous cell population; and 3) compete for nutrients and other environmental resources. Using this assay, we found that in a population of mixed cells grown on 1% ethanol the dominant polygenic trait extending longevity of each of the 3 long-lived yeast mutants decreases the relative fitness of the mutant strain in direct competition with the parental WT strain BY4742. These findings imply that under laboratory conditions that imitate the process of natural selection within an ecosystem composed of yeast cells having different longevity-defining genetic backgrounds, each of the 3 long-lived mutants is forced out of the ecosystem by the parental WT strain exhibiting shorter lifespan. It seems conceivable therefore that 1) yeast cells have evolved some mechanisms for limiting their lifespan upon reaching a certain chronological age; and 2) these mechanisms drive the evolution of yeast longevity towards maintaining a finite yeast lifespan within ecosystems. We speculate that these mechanisms may consist in the ability of the parental WT strain to secrete into growth medium certain compounds (small molecules and/or proteins) that can slow down growth and/or kill long-lived yeast mutants. The challenge for the near future is to identify these compounds responsible for the maintenance a finite yeast lifespan within ecosystems.

## MATERIALS AND METHODS

### Yeast strains and growth conditions

The haploid WT strains BY4741 (*MATa his3D1 leu2D0 met15D0 ura3D0*) and BY4742 (*MATα his3D1 leu2D0 lys2D0 ura3D0*) of the yeast *S. cerevisiae*, the long-lived mutant strains 3, 5 and 12 (each in the BY4742 genetic background), as well as the single-gene-deletion mutant strains *rpp2BΔ* (*MATα his3(1 leu2(0 lys2(0 ura3(0 rpp2B(::kanMX4*) and *dbp3Δ* (*MATα his3(1 leu2(0 lys2(0 ura3(0 dbp3(::kanMX4*) (each in the BY4742 genetic background) were used in this study. All strains were from Open Biosystems. Cells were grown in YP medium (1% yeast extract, 2% peptone) initially containing 0.2% glucose (a fermentable carbon source; CR conditions), 2% glucose (a fermentable carbon source; non-CR conditions), 1% ethanol (a non-fermentable carbon source) or 3% glycerol (a non-fermentable carbon source). Cells were cultured at 30°C with rotational shaking at 200 rpm in Erlenmeyer flasks at a ″flask volume/medium volume″ ratio of 5:1.

### Quantitative mating assay

Cultures of mating type *MATa* (i.e. the haploid WT strain BY4741 [*MATa his3D1 leu2D0 met15D0 ura3D0*]) and mating type *MATα* (i.e. the haploid WT strain BY4742 [*MATα his3D1 leu2D0 lys2D0 ura3D0*] or the selected long-lived haploid mutant strains 3, 5 or 12) were grown separately to mid-logarithmic phase in YP medium (1% yeast extract, 2% peptone) initially containing 0.2% glucose (a fermentable carbon source; CR conditions) or 1% ethanol (a non-fermentable carbon source). Equal numbers (5 × 10^6^) of cells of each mating type were mixed and then collected on a 0.45-μm pore, 25-mm diameter nitrocellulose (NC) filter. The filters were placed on the surface of a YEPD (1% yeast extract, 2% peptone, 2% glucose, 2% agar) plate and incubated at 30°C for 5 hours. The filters were then transferred to Eppendorf tubes and resuspended in 1 ml of a liquid synthetic minimal YNB medium (0.67% Yeast Nitrogen Base without Amino Acids) with 2% glucose. The suspensions were used for making serial 10-fold dilutions. 100-μl aliquots of each dilution were spread on 1) a synthetic minimal YNB medium plate (0.67% Yeast Nitrogen Base without Amino Acids, 2% glucose, 2% agar) without supplements; and 2) a synthetic minimal YNB medium plate supplemented with 20 mg/l *L*-histidine, 30 mg/l *L*-leucine and 20 mg/l uracil. These plates were incubated at 30°C for 2 days. The numbers of diploid cells (*N*_d_) were counted on synthetic minimal YNB medium plates without supplements, whereas the total numbers of cells (*N*_t_) were counted on synthetic minimal YNB medium plates supplemented with 20 mg/l *L*-histidine, 30 mg/l *L*-leucine and 20 mg/l uracil. The efficiency of mating was calculated as the number of colonies of *MATa*/*MATα* diploids (*N*_d_) divided by the sum of *MATa*/*MATα* diploids plus haploid colonies (*N*_t_). Crosses between two WT strains of opposite mating types (i.e. the haploid strain BY4741 [*MATa his3D1 leu2D0 met15D0 ura3D0*]) and the haploid strain BY4742 [*MATα his3D1 leu2D0 lys2D0 ura3D0*]) were used as controls. All tests were carried out in triplicate in 3 independent experiments.

### Quantitative sporulation assay

A small patch of cells of the haploid WT strain BY4741 (*MATa his3D1 leu2D0 met15D0 ura3D0*) was applied to the surface of a master YEPD (1% yeast extract, 2% peptone, 2% glucose, 2% agar) plate. 10^6^ cells of mating type *MATα* (i.e. the haploid WT strain BY4742 [*MATα his3D1 leu2D0 lys2D0 ura3D0*] or the selected long-lived haploid mutant strains 3, 5 or 12) were spread on the surface of a separate crossing plate with YEPD medium. The master plate was replica plated onto a lawn of cells on each of the four crossing plates; different velvet was used for each crossing plate. The crossing plates were incubated overnight at 30°C. Each of the four crossing plates was then replica plated onto a synthetic minimal YNB medium plate (0.67% Yeast Nitrogen Base without Amino Acids, 2% glucose, 2% agar) supplemented with 20 mg/l *L*-histidine, 30 mg/l *L*-leucine and 20 mg/l uracil. These plates were incubated overnight at 30°C. A positive mating reaction between cells of the haploid WT strain BY4741 (*MATa his3D1 leu2D0 met15D0 ura3D0*) and cells of the haploid WT strain BY4742 (*MATα his3D1 leu2D0 lys2D0 ura3D0*) or cells of each of the selected long-lived haploid mutant strains 3, 5 or 12 resulted in confluent growth of diploid cells on a YNB plate (supplemented with *L*-histidine, *L*-leucine and uracil) at the position of a patch of haploid BY4741 cells. To measure sporulation efficiency, cells of each of the four recovered diploid strains were first grown to mid-logarithmic phase in YP medium (1% yeast extract, 2% peptone) initially containing 0.2% glucose (a fermentable carbon source; CR conditions) or 1% ethanol (a non-fermentable carbon source). The cell cycle of these cells was then synchronized by growing them in YPA medium (1% yeast extract, 2% peptone, 2% potassium acetate) from a starting optical density at 600 nm (OD_600_) of 0.2 to final OD_600_ of 1.0; cells were cultured at 30°C with rotational shaking at 200 rpm in Erlenmeyer flasks at a ″flask volume/medium volume″ ratio of 10:1. 2 × 10^7^ of cells from this synchronized culture were then incubated in liquid SPO (0.1% yeast extract, 1% potassium acetate, 0.05% glucose) medium supplemented with 20 mg/l *L*-histidine, 30 mg/l *L*-leucine and 20 mg/l uracil at 30°C for the duration of experiment. At various time points, aliquots of cells were examined for sporulation efficiency by differential interference contrast (DIC) microscopy with an Olympus BX microscope with a ⊆ 100 oil immersion objective. Sporulation efficiency was measured as the percentage of tetrads and dyads produced by a strain, relative to the total number of cells. All tests were carried out in triplicate in 3 independent experiments.

### Cell viability assay for monitoring the susceptibility of yeast to an apoptotic mode of cell death induced by hydrogen peroxide

A sample of cells was taken from a culture at a certain time-point. A fraction of the sample was diluted in order to determine the total number of cells using a hemacytometer. 2 × 10^7^ cells were harvested by centrifugation for 1 min at 21,000 × g at room temperature and resuspended in 2 ml of YP medium containing 0.2% glucose as carbon source. Each cell suspension was divided into 2 equal aliquots. One aliquot was supplemented with hydrogen peroxide to the final concentration of 2.5 mM, whereas other aliquot remained untreated. Both aliquots were then incubated for 2 h at 30°C on a Labquake rotator set for 360° rotation. Serial dilutions of cells were plated in duplicate onto plates containing YP medium with 2% glucose as carbon source. After 2 d of incubation at 30°C, the number of colony forming units (CFU) per plate was counted. The number of CFU was defined as the number of viable cells in a sample. For each aliquot of cells exposed to hydrogen peroxide, the % of viable cells was calculated as follows: (number of viable cells per ml in the aliquot exposed to hydrogen peroxide/number of viable cells per ml in the control aliquot that was not exposed to hydrogen peroxide) × 100.

### Cell viability assay for monitoring the susceptibility of yeast to a liponecrotic mode of cell death induced by palmitoleic acid

A sample of cells was taken from a culture at a certain time-point. A fraction of the sample was diluted in order to determine the total number of cells using a hemacytometer. 2 × 10^7^ cells were harvested by centrifugation for 1 min at 21,000 × g at room temperature and resuspended in 2 ml of YP medium containing 0.2% glucose as carbon source. Each cell suspension was divided into 2 equal aliquots. One aliquot was supplemented with palmitoleic acid (#P9417; Sigma) from a 50 mM stock solution (in 10% chloroform, 45% hexane and 45% ethanol); the final concentration of palmitoleic acid was 0.15 mM (in 0.03% chloroform, 0.135% hexane and 0.135% ethanol). Other aliquot was supplemented with chloroform, hexane and ethanol added to the final concentrations of 0.03%, 0.135% and 0.135%, respectively. Both aliquots were then incubated for 2 h at 30°C on a Labquake rotator set for 360° rotation. Serial dilutions of cells were plated in duplicate onto plates containing YP medium with 2% glucose as carbon source. After 2 d of incubation at 30°C, the number of CFU per plate was counted. The number of CFU was defined as the number of viable cells in a sample. For each aliquot of cells exposed to palmitoleic acid, the % of viable cells was calculated as follows: (number of viable cells per ml in the aliquot exposed to palmitoleic acid/number of viable cells per ml in the control aliquot that was not exposed to palmitoleic acid) × 100.

### Quantifying the relative fitness of a long-lived mutant strain in a direct competition assay with a parental WT strain

The WT strains BY4739 (*MATα leu2D0 lys2D0 ura3D0*) and BY4742 (*MATα his3D1 leu2D0 lys2D0 ura3D0*), the single-gene-deletion mutant strain *dbp3Δ* (*MATα his3(1 leu2(0 lys2(0 ura3(0 dbp3(::kanMX4*) in the BY4742 genetic background (all from Open Biosystems), and the long-lived mutant strains 3, 5 and 12 (all 3 in the BY4742 genetic background) were grown separately in YP medium (1% yeast extract, 2% peptone) initially containing 0.2% glucose, 2% glucose or 1% ethanol as carbon source until mid-exponential phase. Cells were cultured at 30°C with rotational shaking at 200 rpm in Erlenmeyer flasks at a ″flask volume/medium volume″ ratio of 5:1. The single-gene-deletion mutant strain *dbp3Δ* lacks a gene encoding a DEAD-box family protein involved in ribosomal biogenesis [73]. *dbp3Δ* was used as a control mutant strain because it is known to exhibit 1) an extended replicative lifespan (as compared to the parental WT strain BY4742) [73]; 2) a reduced growth rate on 0.2% glucose (this study), 2% glucose [73] and 1% ethanol (this study); and 3) a reduced relative fitness when is co-cultured with a parental WT strain in medium initially containing 2% glucose [73]. 2.5 × 10^6^ cells of the WT strain BY4739 (*MATα leu2D0 lys2D0 ura3D0*) were mixed with the same number of cells of the BY4742 (*MATα his3D1 leu2D0 lys2D0 ura3D0*), *dbp3Δ* (*MAT*α *his3(1 leu2(0 lys2(0 ura3(0 dbp3(::kanMX4*), 3, 5 or 12 strain in 50 ml of YP medium initially containing 0.2% glucose, 2% glucose or 1% ethanol as carbon source. After culturing the cell mixture at 30°C for 7 days, an aliquot of cell suspension was diluted and plated on a solid YP medium supplemented with 2% glucose. Following 2 days of incubation at 30°C, colonies on each plate were replicated onto 2 plates with solid YNB (Yeast Nitrogen Base) medium without amino acids supplemented with 2% glucose; one of these plates contained leucine, lysine, uracil and histidine [hereafter called a ″His^+^″ plate], whereas the other plate contained leucine, lysine and uracil [hereafter called a ″His^−^″ plate]. After 2 days of incubation at 30°C, the number of CFU on ″His^+^″ and ″His^−^″ plates was counted. The relative fitness of each His^+^ strain (relative to the His^−^ strain BY4739 [*MATα leu2D0 lys2D0 ura3D0*]) was calculated as log_2_ [(CFU^x^_mutant_/CFU^x^_WT_/(CFU° _mutant_/CFU° _WT_)], where: CFU^x^ is the colony count at the end of week x, whereas CFU° is the colony count at initial inoculation of a mixed culture.

### Statistical analysis

Statistical analysis was performed using Microsoft Excel's (2010) Analysis ToolPack-VBA. All data on cell survival are presented as mean ± SEM. The *p* values for comparing the means of two groups (using an unpaired two-tailed *t* test) were calculated with the help of the GraphPad Prism statistics software.
